# The Bioinformatics-Based Analysis Identifies 7 Immune-Related Genes as Prognostic Biomarkers for Colon Cancer

**DOI:** 10.3389/fonc.2021.726701

**Published:** 2021-11-26

**Authors:** Jie Pan, Zongqi Weng, Chaorong Xue, Bingqiang Lin, Mengxin Lin

**Affiliations:** ^1^ Department of Emergency Surgery, Fujian Medical University Union Hospital, Fuzhou, China; ^2^ Department of Medical Oncology, Fujian Medical University Union Hospital, Fuzhou, China

**Keywords:** colon cancer (COAD), immune-related gene (IRG), immunotherapy, cancer, genes

## Abstract

Colon cancer poses a great threat to human health. Currently, there is no effective treatment for colon cancer due to its complex causative factors. Immunotherapy has now become a new method for tumor treatment. In this study, 487 DEGs were screened from The Cancer Genome Atlas (TCGA) database and ImmPort database, and GeneOntology (GO) functional enrichment and Kyoto Encyclopedia of Genes and Genomes (KEGG) pathway analysis was performed. Hierarchical clustering of all samples revealed a significant correlation between colon cancer and immunity. The weighted gene co-expression network analysis (WGCNA) algorithm was used to identify key gene modules associated with immunity in colon cancer, here, module grey60 showed the highest correlation. A protein-protein interaction (PPI) network was constructed using the STRING database to screen hub genes, and subsequently, 7 immune-related genes the most closely associated with colon cancer were identified by differential expression in cancer and paracancer. Finally, a risk prediction model was developed using least absolute shrinkage and selection operator (LASSO) COX analysis, and the accuracy of the model was validated by GSE14333. This study determined that IRF4 and TNFRSF17 were immune-related genes in colon cancer, providing immune-related prognostic biomarkers for colon cancer.

## Introduction

Colon cancer (COAD) is a malignant tumor of the gastrointestinal tract. Colon cancer is often classified together with rectal cancer such as colorectal cancer (CRC), which is one of the 5 most frequently diagnosed cancers worldwide, and COAD ranked the 2^rd^ highest cause leading to cancer-related deaths worldwide in 2020 (1). The main risk factors cause COAD are poor dietary behavior, obesity, age, and hereditary mutations (2, 3). Colorectal cancer incidence and mortality are showing an increasing tread, and new causes of colorectal cancer worldwide is expected to reach 2.2 million with 1.1 million deaths by 2030 (4, 5). Colon cancer is a metastatic cancer with common distant metastatic sites in the liver, lung, bone, and brain (6). Patients with early-stage colorectal cancer have a prognostic survival rate close to 90% in contrast to a 5-year survival rate of only 14% for advance-stage patients. In clinical practice, most patient show metastasis by the time diagnosis (7), which is normally associated with a significantly poor prognosis (8). Therefore, developing an effective treatment method for colorectal cancer is currently an urgent task.

In recent years, cancer immunotherapy (CIT) has emerged as a new research hotspot, showing an important link between human immune system and cancer interactions (9). CIT has been implemented to improve the prognosis of patients with malignant solid tumors by mediating tumor destruction through activating anti-tumor immune responses (10, 11). The tumor microenvironment (TME), which plays a crucial role in CIT, consists of immune cells, stromal cells, extracellular matrix, cytokines, and chemokines that could promote immune escape, tumor growth, and metastasis (12, 13). Immune checkpoint inhibitors have been reported to enhance tumor-specific immune responses and reduce immune escape of cancer cells, thereby inhibiting tumor growth (14, 15). Immune-related genes (IRG) have been validated as prognostic biomarkers in non-small cell lung cancer (16), liver cancer (17), clear cell renal cell carcinoma (18), and other cancers.

In this study, immune-related DEGs were screened from the TCGA database and ImmPort database, and the potential functions of DEGs were analyzed using the GO function and KEGG pathway. The WGCNA method was applied to construct a gene co-expression network to find key gene modules significantly associated with colon cancer. A PPI network was constructed to screen hub genes, and a prognostic prediction model was established using LASSO COX analysis. Moreover, the accuracy of the model was verified based on the association between immune-related genes and immune infiltrating cells to provide prognostic biomarkers and potential targets for immunotherapy of COAD.

## Materials and Methods

### Clinical Samples and Data Collection

RNA sequencing (RNA-seq) data and clinicopathological information of 455 colon cancer samples and mRNA data of 41 normal tissues were obtained from the TCGA database(see **Supplementary Table 1** for details). A list of immune-related genes was downloaded from the Immunology Database and Analysis Portal (ImmPort) database (https://www.immport.org) (see **Supplementary Table 2** for details). The score data of the 6 immune-infiltrating cells downloaded from the TIMER database. The expression matrix and clinical information of GSE14333 were acquired from GEO (https://www.ncbi.nlm.nih.gov/geo/) for model verification.

### Differentially Expressed Genes (DEGs) and Functional Enrichment Analysis

Differential analysis of immune-related genes was performed using the R package limma package, with adjust P<0.05, |log2FC| > 1 as the screening conditions, and the genes were shown in volcano plots. Venn diagrams were used to take the intersection of the screened DEGs and immune-related genes. GO functional enrichment and KEGG pathway analysis of the intersected genes were conducted using the R package clusterprofiler package, and adjust P < 0.05 was considered as statistically significant. GSEA was performed between high and low expression of IRF4 and TNFRSF17. NOM p-value <0.05, FDR q-value <0.25, and | NES |> 1 were the threshold.

### Hierarchical Clustering

Consistency analysis was conducted using the R package ConsensusClusterPlus to classify COAD into different subgroups using immune gene set and to observe the relationship between different subgroups and immunity. The maximum number of clusters was 6. The optimal number of clusters was determined by choosing an appropriate K value, and the clustering heat map was analyzed by the R package pheatmap. Heat maps showing the distribution of immune checkpoints in subgroups were plotted by R software ggplot2 and pheatmap.

### Weighted Gene Co-Expression Network Analysis (WGCNA)

WGCNA can predict genes associated with carcinogenesis (Yang et al., 2018). The co-expression network of genes in COAD was constructed using the R package WGCNA to analyze immune-related hub genes and identify gene modules significantly associated with immunity. Soft threshold power (β) was set according to the scale-free topology criterion. The topological overlap matrix dissimilarity was calculated to construct a hierarchical clustering dendrogram to cut a dynamic tree to classify genes with similar expression features and to identify modules. The relationship between co-expression modules and phenotype data was shown by the module-feature relationship heat map, and key gene modules were selected.

### Protein-Protein Interactions (PPI)

The STRING database (http://string-db.org/) is one of the online resources dedicated to the whole-object protein network, which can be used to analyze the functional interaction relationship between proteins (19). The screened modular genes were imported into the STRING database, PPI networks were constructed after isolating nodes and visualized using the MCC (Maximal Clique Centrality) algorithm in cytoHubba, a plugin for cytoscape. The top 10 ranked genes were filtered as hub genes. The expression levels of the 10 hub genes were compared in COAD and paracancer, and the DEGs with significant differences were selected.

### Construction of a Prognostic Feature Model

The relationship between prognostic immune-related gene expression and OS (overall survival) was assessed using LASSO COX analysis. LASSO analysis was performed using the package glmnet. Prognostic risk prediction model for COAD was developed based on LASSO risk score calculation formula. Patients with COAD were divided into high-risk and low-risk groups according to the median risk score. KM curves were plotted to compare the OS between the two risk groups. ROC survival analysis was performed using the R package SURVIVAL, and the “rmda” package was employed for decision curve analysis. The association between the risk score model and tumor immune infiltrating cells was also investigated using spearman correlation analysis. Statistically significant was defined when P<0.05.

## Results

### DEGs in COAD and Immune-Related Genes

**Figure 1:**
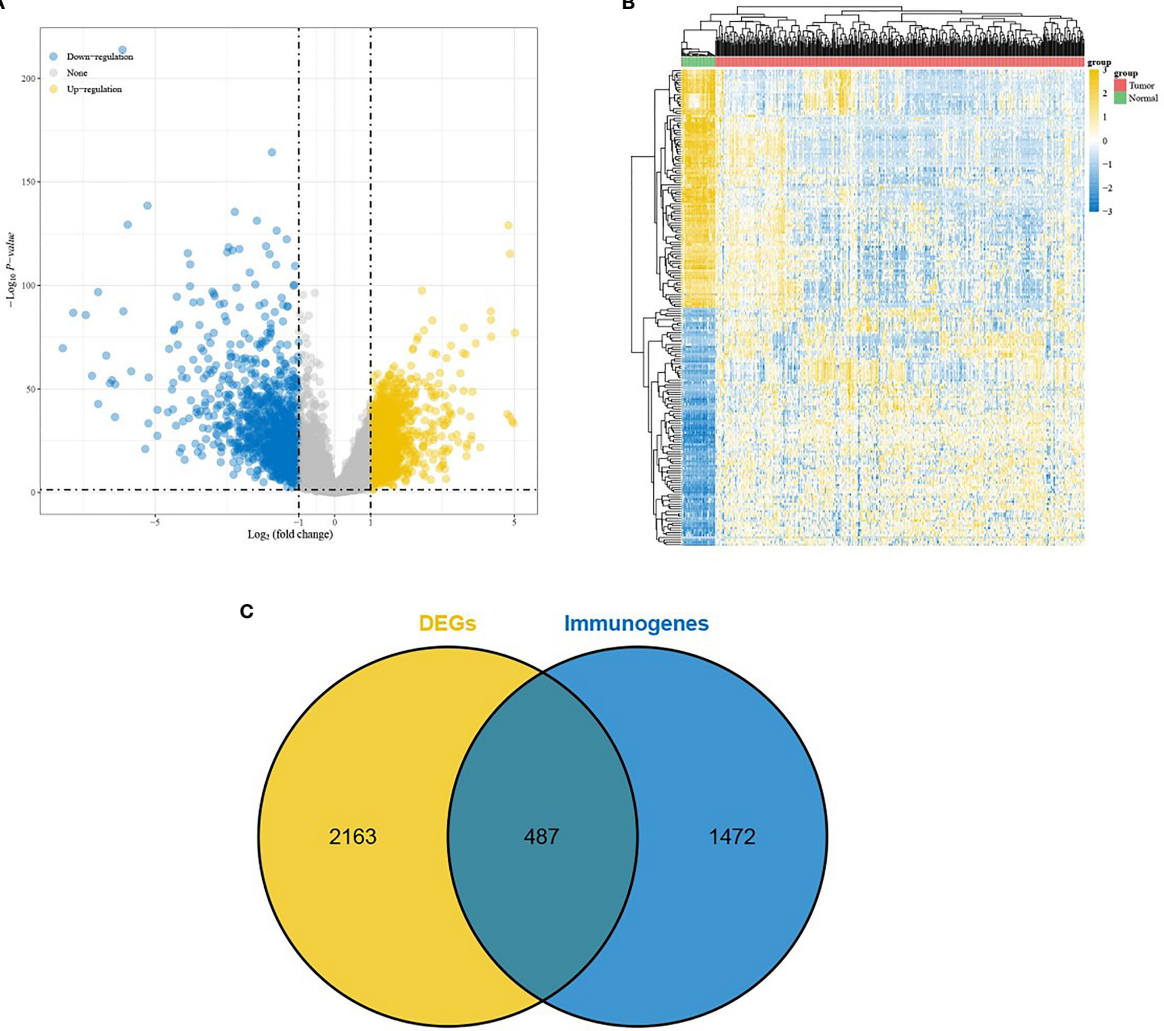
Screening of DEGs. **(A)** Volcano plot and **(B)** Heat map comes from TCGA database; **(C)** Veen plot, taking the intersection of DEGs and immune-related genes.

**Figure 2:**
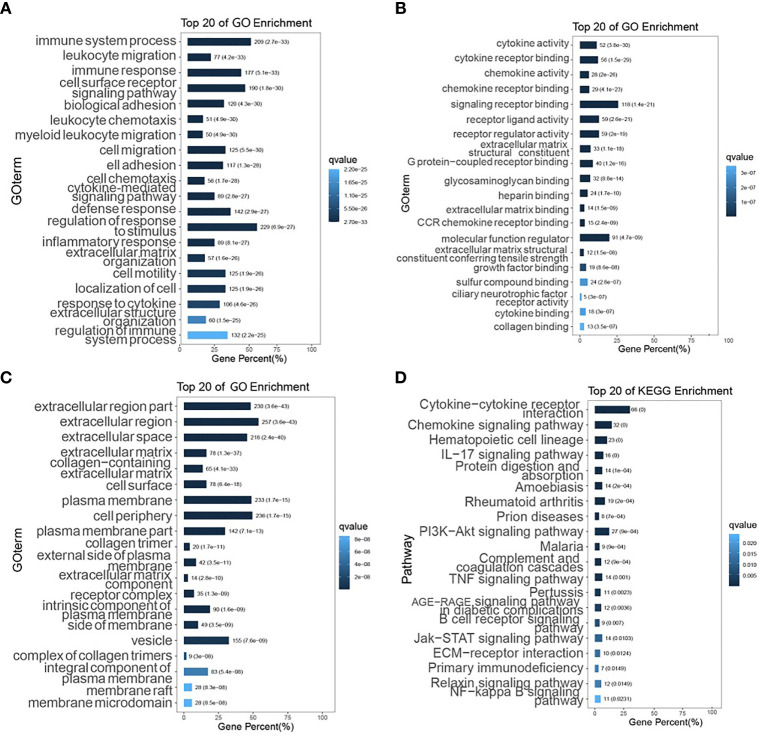
GO function enrichment and KEGG pathway analysis. **(A–C)** The top 20 biological processes, molecular functions, and cellular components of GO terms, respectively; **(D)** The top 20 KEGG pathways.

DEGs were screened from 455 colon cancer tissues and 41 normal tissues in TCGA, and we obtained a total of 1404 up-regulated genes and 1246 down-regulated genes(see **Supplementary Table 3** for details). Volcano and heat maps are shown in **Figures 1A, B**, respectively. 487 overlapping DEGs were identified using venn plots to take the intersection of the screened DEGs and immune gene sets (**Figure 1C**). GO function enrichment and KEGG difference analysis on overlapping DEGs revealed that in immune system processes, the immune response was the most significant biological process (BP), cytokine activity was the most significant molecular function (MF), the extracellular region was the most significant cellular composition (CC), cytokine-cytokine receptor interaction was the most significant KEGG pathway (**Figures 2A**–**D**). **Tables 1** and **2** display the top 20 GO functions and KEGG pathways, respectively.

**Table 1:** Top 20 GO terms.

Description	Class	Pvalue	Qvalue
extracellular region part	Cellular Component	7.13E-46	3.59E-43
extracellular region	Cellular Component	1.18E-45	3.59E-43
extracellular space	Cellular Component	1.17E-42	2.38E-40
extracellular matrix	Cellular Component	8.43E-40	1.28E-37
immune system process	Biological Process	4.67E-37	2.73E-33
leukocyte migration	Biological Process	1.45E-36	4.23E-33
immune response	Biological Process	2.62E-36	5.10E-33
collagen-containing extracellular matrix	Cellular Component	3.32E-35	4.06E-33
cell surface receptor signaling pathway	Biological Process	1.22E-33	1.78E-30
biological adhesion	Biological Process	3.70E-33	4.32E-30
cytokine activity	Molecular Function	4.00E-33	3.76E-30
leukocyte chemotaxis	Biological Process	5.65E-33	4.93E-30
myeloid leukocyte migration	Biological Process	5.90E-33	4.93E-30
cell migration	Biological Process	7.58E-33	5.54E-30
cytokine receptor binding	Molecular Function	3.13E-32	1.47E-29
cell adhesion	Biological Process	2.03E-31	1.32E-28
cell chemotaxis	Biological Process	2.89E-31	1.69E-28
cytokine-mediated signaling pathway	Biological Process	5.35E-30	2.84E-27
defense response	Biological Process	5.87E-30	2.86E-27
regulation of response to stimulus	Biological Process	1.53E-29	6.86E-27

**Table 2:** Top 20 KEGG pathways.

Pathway	Pvalue	Qvalue
Cytokine-cytokine receptor interaction	5.63E-36	1.24E-33
Chemokine signaling pathway	3.77E-14	4.17E-12
Hematopoietic cell lineage	5.29E-09	3.90E-07
IL-17 signaling pathway	1.64E-07	9.04E-06
Protein digestion and absorption	2.82E-06	1.25E-04
Amoebiasis	5.05E-06	1.86E-04
Rheumatoid arthritis	6.33E-06	2.00E-04
Prion diseases	2.71E-05	7.48E-04
PI3K-Akt signaling pathway	4.23E-05	9.42E-04
Malaria	4.60E-05	9.42E-04
Complement and coagulation cascades	4.69E-05	9.42E-04
TNF signaling pathway	5.33E-05	9.82E-04
Pertussis	0.00013267	2.26E-03
AGE-RAGE signaling pathway in diabetic complications	0.00022641	3.57E-03
B cell receptor signaling pathway	0.000475706	7.01E-03
Jak-STAT signaling pathway	0.000744929	1.03E-02
ECM-receptor interaction	0.000957602	1.24E-02
Primary immunodeficiency	0.001236546	1.49E-02
Relaxin signaling pathway	0.001282748	1.49E-02
NF-kappa B signaling pathway	0.002089419	2.31E-02

### COAD Subgroups Were Significantly Associated With Immunity

**Figure 3:**
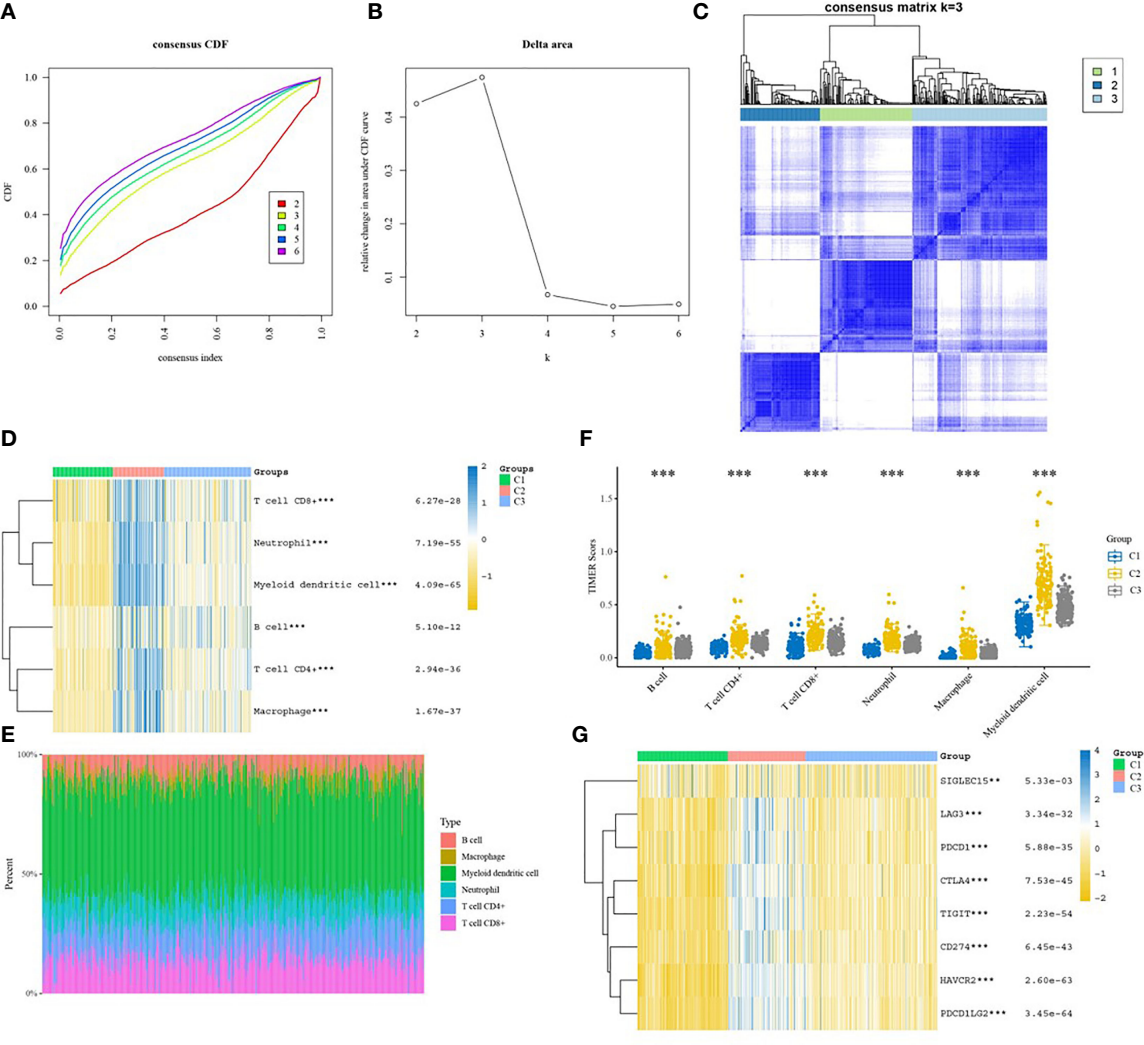
Immunoclustering analysis of colon cancer subgroups. **(A)** CDF graph; **(B)** CDF Delta area graph; **(C)** Consensus matrix heat map of the three sample clusters defined with a consensus range of 0-1, with 0 representing white, meaning that the samples do not cluster, and 1 representing blue, meaning that the samples always cluster; **(D)** Heat map of the distribution of immune cells in different subgroups; **(E)** Percentage abundance of tumor-infiltrating immune cells in each sample, with different colors representing different immune cell types, horizontal coordinates represent samples, vertical coordinates represent the percentage of immune cell content in individual samples; **(F)** TIMER score distribution of 6 infiltrating species immune cells in different subgroups; **(G)** Heat map of the distribution of immune checkpoint genes in different subgroups. *p < 0.05, **p < 0.01, ***p < 0.001.

Hierarchical clustering was performed on all samples (**Figures 3A, B**). As shown in **Figure 3C**, the consensus matrix heat map was more neatly stratified when the optimal number of clusters k=3, which indicated a better reliability and stability when the samples were divided into 3 subgroups. The relationship between the 6 immune infiltrating cells and each subgroup was presented in the heat map (**Figure 3D**), and the percentage abundance of each cluster is shown in **Figure 3E**, from which myeloid dendritic cells showed the highest content. **Figure 3F** shows the distribution of the scores of the 6 immune infiltrating cells in the 3 subgroups. Kruskal-Wallis was used to analyze the expression distribution of immune checkpoint genes in the three subgroups. Each gene was significantly expressed, and its expression was up-regulated in group2 and down-regulated in the other two groups (**Figure 3G**).

### WGCNA Identified Key Modules for COAD

**Figure 4:**
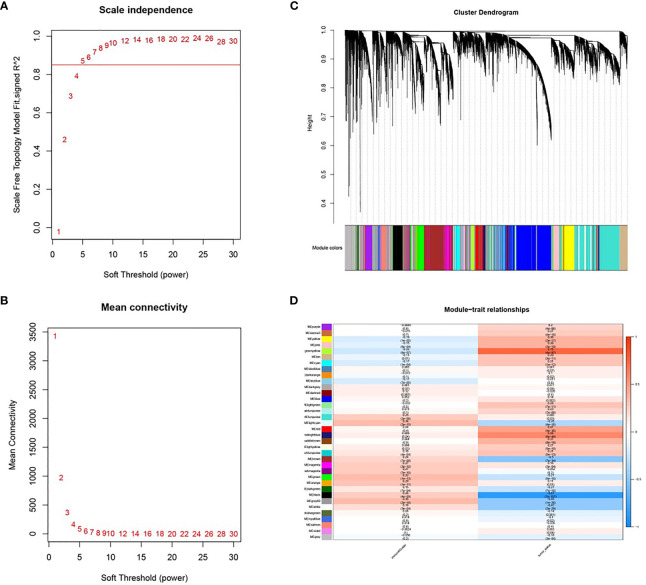
Co-expression modules based on WGCNA screening. **(A, B)** Soft threshold screening (β=5); **(C)** Clustering dendrogram and module colors; **(D)** Heat map of correlation between module feature genes and clinical features, columns represent colors and rows represent clinical features.

A soft threshold of β=5 was chosen to create a scale-free topological network (**Figures 4A, B**). A total of 36 gene modules were generated and expression was shown using different colors (**Figure 4C**). The number of genes contained in each module is presented in **Table 3**. Among them, the grey60 module (R=0.35, P=2e-16) was the most closely associated with tumor immunity, therefore it was considered as the key gene module (**Figure 3D**).

**Table 3:** Gene module colors and corresponding numbers.

Colors	Number	Colors	Number
black	735	lightcyan	315
blue	3327	lightgreen	189
brown	2134	orange	88
cyan	378	paleturquoise	51
darkgreen	114	pink	721
darkgrey	90	purple	572
darkmagenta	38	red	777
darkolivegreen	39	royalblue	174
darkorange	78	saddlebrown	55
darkred	157	salmon	386
darkturquoise	111	sienna3	35
green	868	skyblue	56
greenyellow	546	steelblue	52
grey	2129	tan	534
grey60	218	turquoise	3819
lightyellow	176	violet	40
magenta	631	white	70
midnightblue	337	yellow	873
nodeAttr[nodesPresent]	1		

### PPI Network Construction

**Figure 5:**
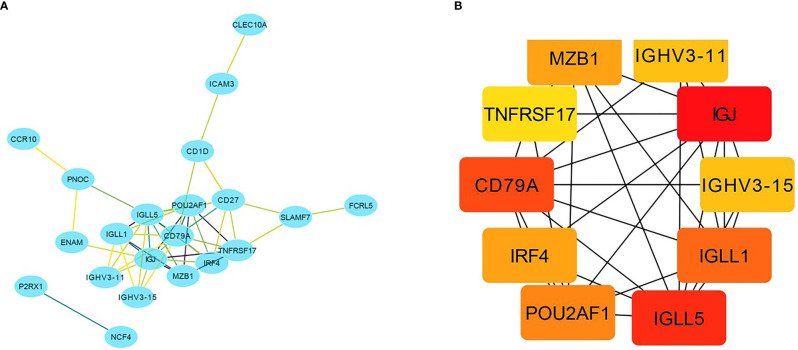
Important gene module and hub gene screening. **(A)** Hub genes identified in the grey60 module; **(B)** Top 10 hub genes screened in the grey60 module.

**Figure 6:**
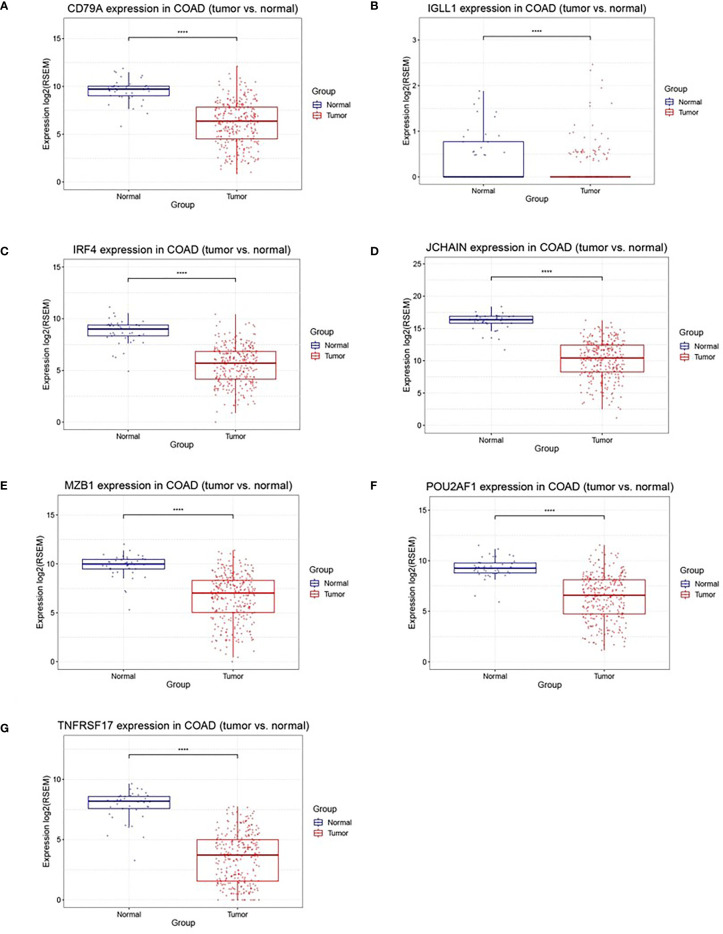
Differently expression of 7 hub genes between cancer tissues and normal tissues in colon cancer. **(A–G)** were CD79A, IGLL1, IRF4, JCHAIN (IGJ), MZB1, POU2AF1, TNFRSF17, respectively, ****P < 0.001.

A total of 218 genes in the key gene module grey60 were imported into the STRING database to obtain their interactions (**Figure 5A**). The 10 top hub genes were screened (**Figure 5B**), and the score of each gene was displayed in **Table 4**, from which IGJ (JCHAIN) showed the highest score. Subsequent identification of the hub genes revealed that 7 genes were significantly different in COAD *versus* paracancer and all their expression was down-regulated (**Figure 6**).

**Table 4:** Gene scores of top 10.

Rank	Name	Score
1	IGJ (JCHAIN)	133
2	IGLL5	127
3	CD79A	112
4	IGLL1	96
5	POU2AF1	84
6	MZB1	30
6	IRF4	30
8	IGHV3-11	24
8	IGHV3-15	24
10	TNFRSF17	16

### Immune-Related Genes Were Associated With Prognosis

**Figure 7:**
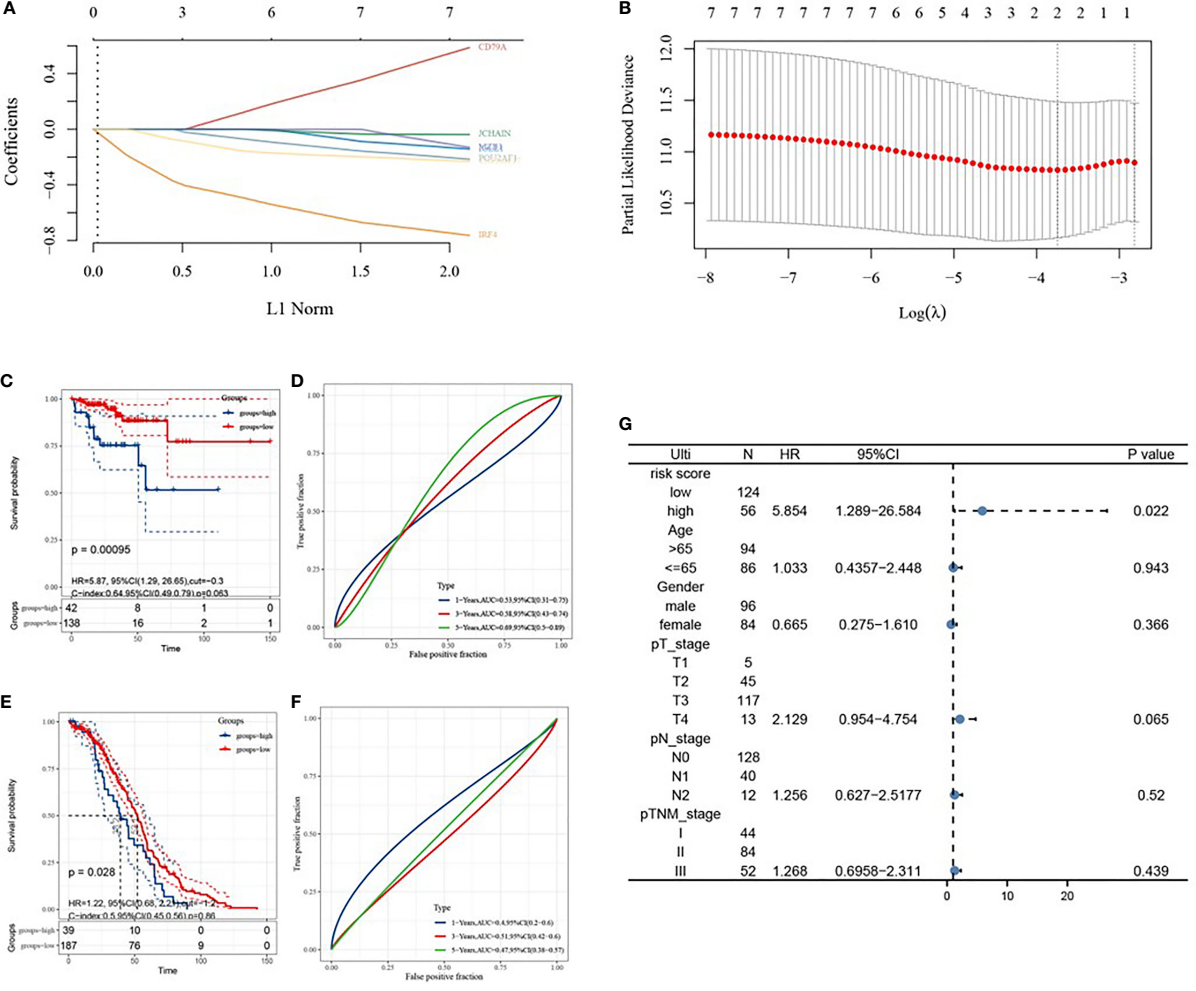
Construction of prognostic features of immune-related genes in colon cancer. **(A, B)** distribution of LASSO coefficients for 7 hub genes to obtain the adjustment parameter λ.min=0.0075, and the vertical black dashed line in B defines the optimal λ value; **(C)** Distribution of risk scores of colon cancer patients; **(D)** KM curve of high and low risk group; **(E)** ROC curve; **(F)** Univariate risk proportional regression model; **(G)** KM curve of high and low risk group, from GSE14333; **(H)** 1-year, 3-year, and 5-year ROC curves, from GSE14333.

Based on LASSO Cox analysis, a DFS prognostic feature model was established using the 7 IRGs (**Figures 7A, B**). When λ _min_=0.0236, a prognostic model containing two genes was obtained. Riskscore=(-0.3063)*IRF4+(-0.0477)*TNFRSF17 was employed to calculate the risk score value of each sample, and divide all the samples into high-risk groups and low-risk groups with the best cut-off value (cut=-0.3). The KM curve showed the difference in survival between the two risk groups (P=0.00095), and the ROC curve showed the predictive ability of the model (**Figures 7C, D**). GSE14333 served as a validation set to classify the samples according to the best cutoff value (cut=-1.2), and draw the KM curve and ROC curve validation model (**Figures 7E, F**). Univariate COX analysis showed that the risk score was statistically significant (P=0.022).

**Figure 8:**
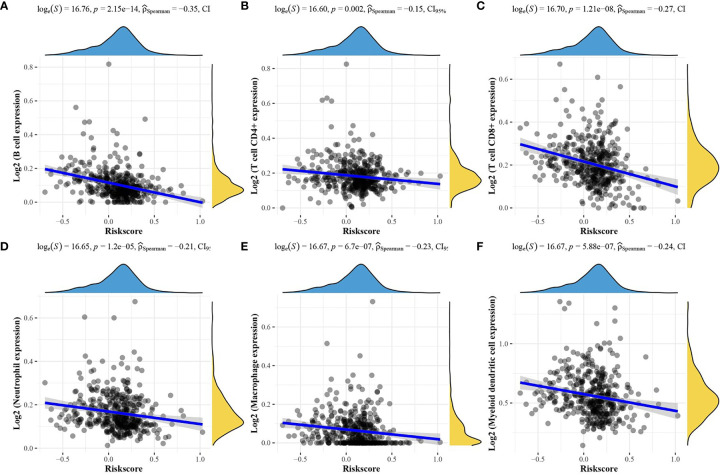
Relationship between the risk score model and the abundance of the six immune infiltrating cells. **(A)** B cells; **(B)** CD4+ T cells; **(C)** CD8+ T cells; **(D)** neutrophils; **(E)** Macrophages, **(F)** Myeloid dendritic cells.

To verify whether the risk score model could reflect the status of the tumor immune microenvironment in patients, the relationship between the risk score model and immune infiltrating cells was investigated, as shown in **Figures 8A–F**. All the 6 types of immune infiltrating cells were found to be negatively correlated with the risk score.

### IRF4 and TNFRSF17 Participated in the Immune Response of COAD

**Figure 9:**
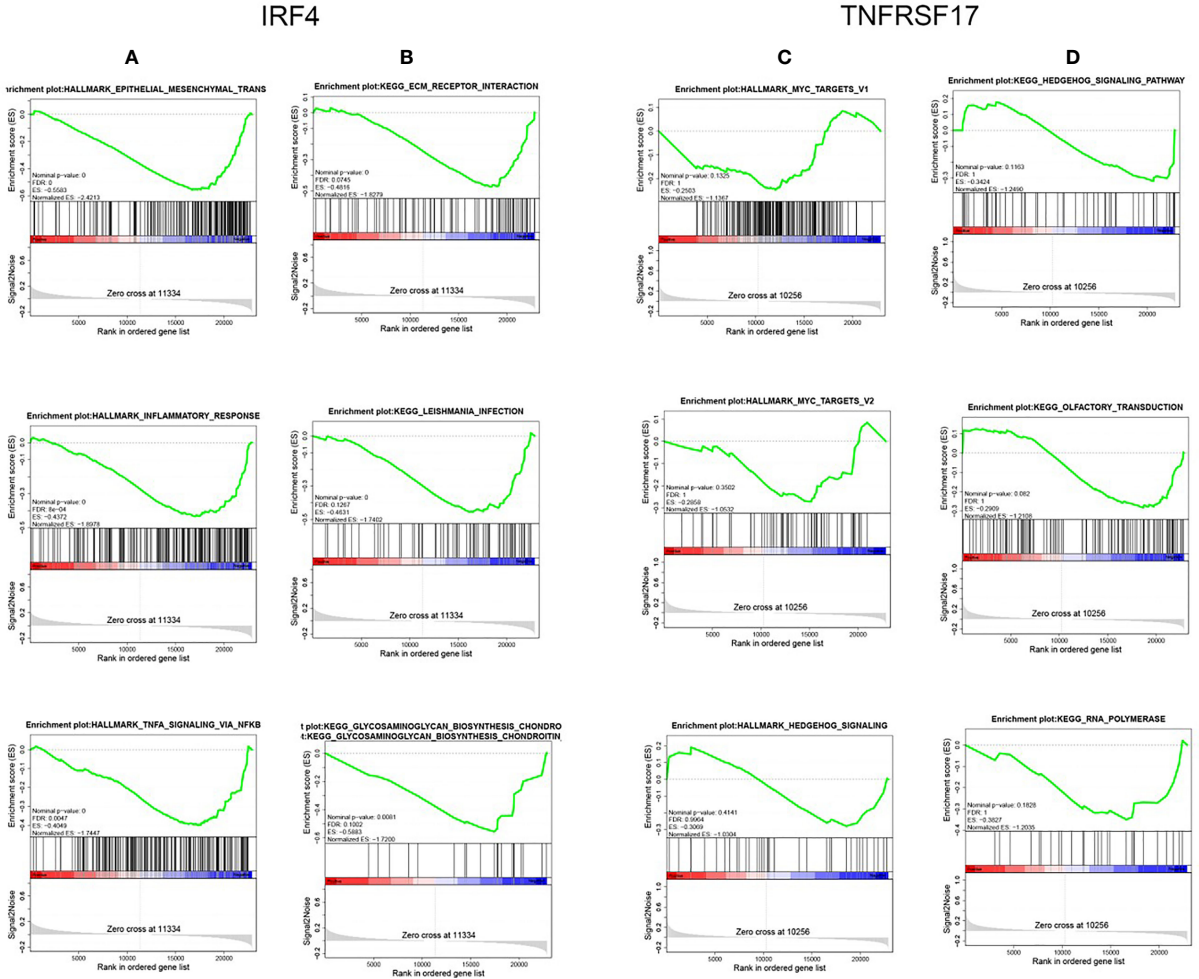
GSEA. Top 3 Hallmark and KEGG pathways of IRF4 and TNFRSF17, respectively. **(A, C)** are Hallmark pathways, and **(B, D)** are KEGG pathways.

To understand the specific biological pathways of IRF4 and TNFRSF17, we conducted GSEA to explore their enriched gene sets in different subgroups. The top 10 KEGG pathways and hallmarks of each gene are shown in **Tables 5** and **6**. When IRF4 and TNFRSF17 were low-expressed and were significantly enriched in immune-related pathways (**Figure 9**), such as receptor interaction, inflammatory response, and hedgehog signaling pathway. The results indicated that IRF4 and TNFRSF17 were involved in the immune response and may be potential indicators for COAD immunotherapy.

**Table 5:** The top 10 Hallmark and KEGG pathway of IRF4.

	Name	Size	ES	NES	NOM p-val	FDR q-val
KEGG	ECM_RECEPTOR_INTERACTION	83	-0.482	-1.828	0	0.075
	LEISHMANIA_INFECTION	69	-0.463	-1.740	0	0.127
	GLYCOSAMINOGLYCAN_BIOSYNTHESIS_CHONDROITIN_SULFATE	22	-0.588	-1.72	0.008	0.100
Hallmark	EPITHELIAL_MESENCHYMAL_TRANSITION	197	-0.558	-2.421	0	0
	INFLAMMATORY_RESPONSE	198	-0.437	-1.898	0	0.001
	TNFA_SIGNALING_VIA_NFKB	196	-0.405	-1.745	0	0.005

**Table 6:** The top 10 Hallmark and KEGG pathway of TNFRSF17.

	Name	Size	ES	NES	NOM p-val	FDR q-val
KEGG	HEDGEHOG_SIGNALING_PATHWAY	53	-0.342	-1.249	0.116	1
	OLFACTORY_TRANSDUCTION	111	-0.291	-1.211	0.082	1
	RNA_POLYMERASE	28	-0.383	-1.204	0.183	1
Hallmark	MYC_TARGETS_V1	194	-0.250	-1.137	0.133	1
	MYC_TARGETS_V2	57	-0.286	-1.053	0.350	1
	HEDGEHOG_SIGNALING	36	-0.307	-1.030	0.414	0.996

## Discussion

Tumor microenvironment has been increasingly important in cancer treatment. Tumor microenvironment consists of tumor cells and surrounding non-tumor cells, such as immune cells and fibroblasts (20). The composition of tumor immune cells is fundamental in determining the fate of tumors and their ability to invade and metastasize (21). Previous studies found that immune cells with different compositions behaves differently in colorectal cancer and normal intestinal tissues and differs at different stages of the tumor (22). Despite the advances in the treatment modalities of COAD, there is still an urgent need to address the issues related to immunotherapy of the cancer. The aim of this study was to identify immune-related genes in COAD and to develop a prognostic risk score model and validate its accuracy.

Immune-related genes play an important role in tumor immunotherapy. As immune-related genes can be quantified in multiple cell types, their expression could serve as a better tumor biomarker (23). In this study, an initial screening of immune-related genes in COAD was performed using the TCGA database and the ImmPort database. Subsequently, the function and pathway enrichment analysis of DEGs found that the molecular function was significantly enriched in cytokine activity, and it was significantly related to the cytokine-cytokine receptor interaction pathway. Cytokines are products of immune cells that regulate the proliferation, differentiation, effector functions, and survival of leukocytes, and have the ability to enhance immune responses and destroy cancer cells (24, 25). It has been shown that cytokines may be associated with tumor aggressiveness (26) and could directly or indirectly inhibit tumor cell growth (27). Cytokines also play important role in COAD, for example, interleukin-34 (IL-34) expression is upregulated in colorectal cancer and promotes cancer cell growth (28); interleukin-23 (IL-23)-induced immune cell activation exacerbates intestinal inflammation and promotes COAD growth (29).

Tumor microenvironment can lead to increased systemic inflammatory responses and oxidative stress fibrosis, and will affect cancer treatment and prognosis through its participation in metastasis, immune infiltration, some other pathways and functions of cancer cells (30, 31). In recent years, the association between tumor immune infiltrating cells and cancer has received much attention from scholars. In this study, we performed stratified clustering on COAD and analyzed the relationship between the cancer and immunity. The results showed that COAD was significantly associated with immune infiltrating cells, which was consistent with the existing findings (32). Subsequently, 7 immune-related genes of COAD (CD79A, IGLL1, IRF4, JCHAIN, MZB1, POU2AF1, TNFRSF17) were screened by WGCNA algorithm and PPI network construction. Immune-related genes can promote tumor cell proliferation, invasion, and migration (33) and are associated with prognosis (34, 35). In non-squamous non-small cell lung cancer and papillary thyroid cancer, immune-related genes demonstrated its prognostic significance (16, 36). Prognostic prediction models have been developed based on immune infiltration for COAD (37). Also, prognostic correlation between COAD with different TNM stages and immune-related genes has been investigated using COX regression analysis (22). In this study, LASSO COX analysis was applied to establish a prognostic risk score model for COAD based on immune-related genes, and two prognostic signatures (IRF4, TNFRSF17) were obtained. The GSE14333 verification showed that the prognosis prediction model was relatively accurate, and the two prognostic signatures were significantly related to poor prognosis of the cancer. GSEA results demonstrated that a low expression of IRF4 and TNFRSF17 is related to the immune response signaling pathway.

In conclusion, this study revealed the pathways of immune-related genes in the tumor microenvironment of COAD through bioinformatics analysis, and finally identified 7 immune-related genes the most closely associated with COAD development and progression. These findings provide immune-related prognostic biomarkers for COAD and provide effective targets for the clinical treatment of the cancer.

## Data Availability Statement

The original contributions presented in the study are publicly available. This data can has been deposited to NCBI [accession number: PRJNA303175 and GSE14333].

## Author Contributions

All authors listed have made a substantial, direct, and intellectual contribution to the work, and approved it for publication.

## Funding

This work was supported by Joint Funds for the innovation of science and Technology, Fujian province 2019Y9081.

## Conflict of Interest

The authors declare that the research was conducted in the absence of any commercial or financial relationships that could be construed as a potential conflict of interest.

## Publisher’s Note

All claims expressed in this article are solely those of the authors and do not necessarily represent those of their affiliated organizations, or those of the publisher, the editors and the reviewers. Any product that may be evaluated in this article, or claim that may be made by its manufacturer, is not guaranteed or endorsed by the publisher.
